# HIV-1 Viral Load Assays for Resource-Limited Settings

**DOI:** 10.1371/journal.pmed.0030417

**Published:** 2006-10-10

**Authors:** Susan A Fiscus, Ben Cheng, Suzanne M Crowe, Lisa Demeter, Cheryl Jennings, Veronica Miller, Richard Respess, Wendy Stevens, the Forum for Collaborative HIV Research Alternative Viral Load Assay Working Group

## Abstract

The authors discuss studies on the low-cost viral load assays that are currently available and their potential for use in resource-limited settings.

Tremendous strides have been made in treating HIV-1 infection in industrialized countries. Combination therapy with antiretroviral (ARV) drugs suppresses virus replication, delays disease progression, and reduces mortality. In industrialized settings, plasma viral load assays are used in combination with CD4 cell counts to determine when to initiate therapy and when a regimen is failing. In addition, unlike serologic assays, these assays may be used to diagnose perinatal or acute HIV-1 infection.

Unfortunately, the full benefits of antiretroviral drugs and monitoring tests have not yet reached the majority of HIV-1–infected patients who live in countries with limited resources. In this article we discuss existing data on the performance of alternative viral load assays that might be useful in resource-limited settings. Our search strategy and selection criteria for relevant studies are shown in Box 1.

## Viral Load Assays Currently Used in Developed Countries

Currently there are three HIV-1 RNA assays licensed by the United States Food and Drug Administration: Roche Amplicor HIV-1 Monitor Test, version 1.5, bioMérieux NucliSens HIV-1 QT Assay, and Versant HIV-1 RNA 3.0 Assay (bDNA). These assays have been previously described in detail ([1–7], Table 1) and are validated, undergo quality control by the manufacturers, recognize most HIV-1 subtypes, and are familiar to many clinicians.

**Table 1:**
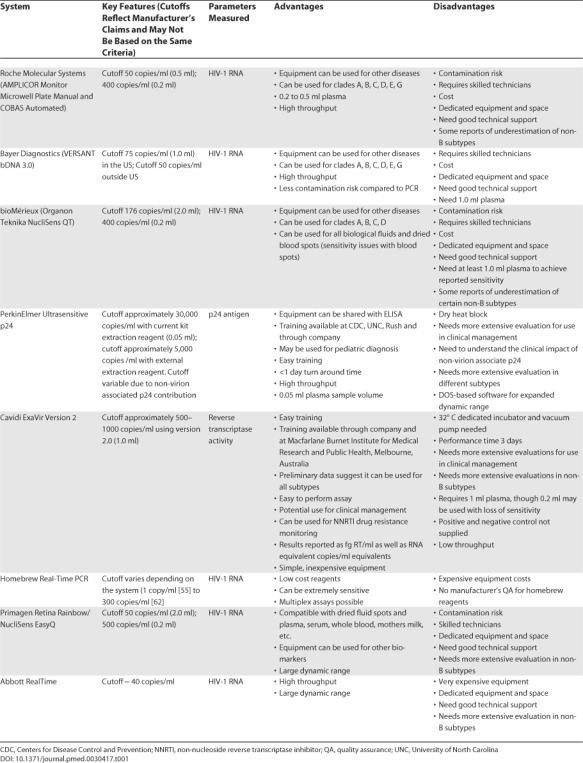
Summary of Commercially Available Viral Load Assays

However, these kits are expensive (kit cost of $50–$100/test in the US), and rely on expensive, often dedicated equipment that can only be used for that assay. Although the manufacturers are decreasing kit costs and in some instances equipment costs for resource-limited countries, these assays are still technologically complex and require physical resources, such as uninterrupted electricity, air conditioning, and access to clean water, that may not be available in less-developed countries (see Figure 1) [8]. Newer assays making use of real-time polymerase chain reaction (Roche TaqMan, Abbott RealTime) or molecular beacon technology (Retina Rainbow, NucliSens EasyQ) are also available, but have not yet been approved by the Food and Drug Administration, and very few papers have been published describing their use.

**Figure 1:**
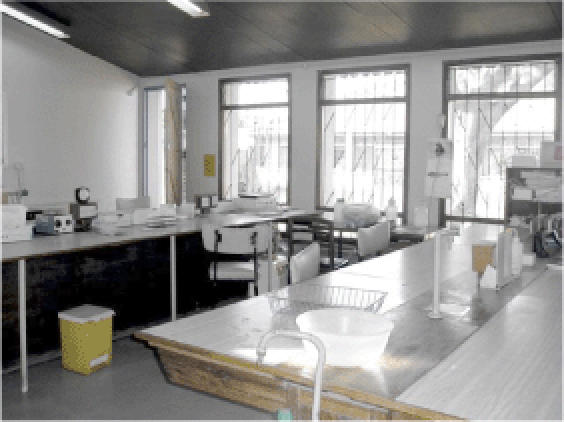
A Laboratory in Malawi Many laboratories in low-income settings, such as the laboratory shown here, are poorly resourced. (Photo: Susan Fiscus)

## Barriers to Using Current Viral Load Assays in Resource-Limited Settings

Resources vary significantly by country. Human resources and infrastructure that might be readily available in major cities, such as trained personnel, clean water, and electricity, may not be found in more rural areas. Reference laboratories that have the personnel, equipment, and infrastructure to perform CD4 cell counts and viral load testing may lack the resources to purchase the kits. Thus, what works for one country, or even for one city within a country, may not apply in all resource-limited settings. Options available to developing countries include performing the laboratory testing at the local site or transporting the specimens to the reference laboratories for testing. A well-defined infrastructure is needed to ensure proper specimen handling and efficient results reporting.

While viral load kit prices have been decreasing for resource-poor countries, the cost of assay disposables and the establishment of a solid infrastructure still limit the utility of HIV-1 RNA for monitoring ARV therapy in most resource-limited settings. Inexpensive, technologically simpler assays are still needed [9–11]. Table 1 summarizes characteristics of some alternative assays, and a more detailed description, together with a summary of the studies on assay performance, is provided below.

## Ultrasensitive P24 Antigen Assay (PerkinElmer Life and Analytical Sciences)

The ultrasensitive p24 (Ultra p24) antigen assay uses a standard ELISA (enzyme-linked immunosorbent assay) format for the capture and detection of HIV-1 p24 antigens coupled with a specific amplification process to increase the assay sensitivity. Heat denaturation of the plasma prior to binding of p24 antigen in the ELISA step helps dissociate immune complexes and denature the antibodies so they no longer compete for binding to the p24 antigen. The dynamic range of the assay can be increased by using a kinetic read-out with the Quanti-Kin Detection System Software [12]. An early version of the Ultra p24 antigen assay was first described by Jorg Schupbach in 1993 using the PerkinElmer HIV-1 p24 antigen kit [13]. Optimization of the standard p24 antigen assay by Schupbach [14–22] includes an external reagent that improves the antigen detection sensitivity, perhaps by greater dissociation of the immune complexes [21]. A number of investigators around the globe have evaluated the assay, with mixed results.

## Viral Load Monitoring Using the Ultrasensitive P24 Antigen Assay

Schupbach and colleagues have reported that the HIV-1 p24 antigen typically decreases in parallel with HIV-1 RNA in successfully treated patients [17] and is a good prognostic indicator of disease progression [17,20], at least among those with HIV-1 subtype B infection. P24 antigen detection is a significant inverse correlate of CD4 cell changes in virally suppressed patients [21] as well as in patients studied longitudinally who were either treatment naïve (85% of the population) or treated with dual nucleoside reverse transcriptase inhibitors (15%) [20] or were on a structured treatment interruption study [22].

Some investigators have been able to produce similar results when monitoring infected patients [23–26] while others have had more disappointing results [27–29]. Pascual et al. [23] compared the Roche RNA assay to the Ultra p24 antigen assay in 130 patients from the US and Malawi (subtypes B and C) using the kit buffer. The RNA assay was more sensitive in detecting HIV-1 when compared to the Ultra p24 antigen assay (95.4% vs. 84.6%). The Ultra p24 antigen assay detected 66.7% of specimens with viral loads of less than 10,000 copies/ml, 87% of specimens with viral loads between 10,000 and 100,000 copies/ml, and 97.7% of specimens with viral load greater than 100,000 copies/ml. Similar results were observed in Burkina Faso [24] using the kit buffer where the Ultra p24 antigen assay detected 27%, 80%, and 87% of samples with viral loads <1000, 1000–63,000, and >80,000 copies/ml, respectively. Better results were observed with subtype C clinical specimens where 95%–100% of samples with detectable viral loads over 400 copies/ml had detectable p24 antigen levels [23,25].

**Box 1. Search Strategy and Selection Criteria**

Members of the Forum for Collaborative HIV Research Alternative Viral Load Assay Working Group include leading academic investigators in this field as well as representatives from the US Centers for Disease Control and Prevention, the National Institutes of Health, charitable organizations that fund validation studies of the assays, and industrial sponsors. We included all peer-reviewed published papers found in PubMed using the search terms: “Alternative HIV-1 viral load assays,” “HIV-1 viral load AND resource-limited settings,” “real-time HIV-1 PCR,” “p24 antigen,” “Cavidi,” “TaqMan,” “EasyQ,” “Retina Rainbow,” and “Abbott RealTime.”

In South Africa, Stevens and colleagues [25] monitored 20 patients (subtype C) who were being treated with highly active antiretroviral therapy. In 19 of 20 patients the Ultra p24 antigen serial results (external buffer) paralleled the RNA results. Ribas and colleagues [26] evaluated 40 patients of different HIV-1 subtypes being treated with ARV therapy. In 33 patients, the changes in p24 antigen (external buffer) correlated well with RNA results, while seven others showed a discrepancy. Since the seven patients with discrepancies during monitoring were infected with seven different subtypes and other patients infected with the same subtypes did not show discrepancies, it is unlikely that the observed differences were related to virus subtype.

In contrast, Prado and colleagues [27] monitored patients in Spain undergoing structured treatment interruption and found little correlation between HIV-1 RNA and the Ultra p24 assay using the kit buffer. In this study, 76% of the treated patients and 49% of the naïve patients showed discordance between HIV-1 RNA detection and p24 detection after viral rebound. Bonard and colleagues [28] tested plasma specimens from 14 patients treated with highly active antiretroviral therapy in Côte d’Ivoire and found weaker changes during treatment with the Ultra p24 antigen assay using the kit buffer compared to the HIV-1 RNA results. Seven of the 14 patients had a dramatic decrease in HIV-1 RNA, but only five of these had similar, though less dramatic, changes in p24 antigen. Of the seven who failed ARV treatment, only three had p24 antigen results that seemed to mirror the RNA results. Use of the external buffer [21] has improved the sensitivity of the assay 2- to 5-fold as well as the correlation with HIV-1 RNA (r = 0 .74 vs. 0.61) [30].

## Infant Diagnosis Using the Ultrasensitive P24 Antigen Assay

Diagnosis of perinatal HIV infection is hindered by the presence of maternal immunoglobulins, which can persist in the infant for as long as 15–18 months. Thus, routine antibody assays cannot be used to diagnose infection until after maternal antibodies have waned.

In industrialized countries, infants are typically diagnosed within days or weeks of birth by confirmed, repeated detection of HIV-1 DNA or RNA [31–35]. However, given the expense and complexity of nucleic acid testing, the World Health Organization strongly encourages the development of technologically simpler, less expensive assays that can be used to diagnose HIV-1 infection in early infancy [36]. The Ultra p24 assay is gaining support as a tool for detection of HIV-1 infection in infants following mother-to-child transmission. Lyamuya and colleagues [37] showed that even an early version of the heat-denatured p24 antigen assay was 99% sensitive and 100% specific in diagnosing HIV-1 subtypes A and D infection in children in Tanzania. Similar results have been observed with subtype B in Switzerland where the sensitivity was 100% after day 10 of age and specificity was 93.8% (99.2% after neutralization) [38], subtype E in Thailand (sensitivity 100%, specificity 100%) [39], subtype C (sensitivity 97%–98%, specificity 97%–99%) [40,41], and multiple subtypes from the Democratic Republic of Congo using either plasma (sensitivity 92.3%, specificity 100%) or dried plasma spots (sensitivity and specificity 100%) [42]. Others have also modified the assay for use with dried plasma spots [43,44], although only one has been successful in adapting the assay to dried blood spots [45].

## Cavidi ExaVir Load Reverse Transcriptase Assay

The reverse transcriptase (RT) enzyme is extracted from the virus particle in the Cavidi RT assay using a solid phase extraction manifold, and is quantified in a functional assay whereby RT synthesizes BrdU-DNA from a poly-A template bound to a 96-well plate [46]. Synthesized DNA is then quantified using anti-BrdU conjugated to alkaline phosphatase followed by the addition of its substrate. The RT activity in the unknown sample is compared to that of a recombinant RT enzyme standard with a known concentration. The extrapolated result is reported as fg RT/ml of plasma or as HIV-1 RNA equivalents/ml using a conversion factor supplied by the manufacturer. The assay has recently undergone revisions to improve sensitivity [47]. Since the assay measures a virion-associated enzyme, results are usually more comparable to plasma RNA [48]. Additionally, since this is a functional assay for RT and does not rely on specific protein or nucleic acid sequences, it performs well when quantifying any HIV-1 subtype [49–51]. A potential concern is that viruses with heavily mutated RT enzymes may be underestimated when compared to a recombinant wild-type RT enzyme, although preliminary studies suggest this may not be a significant problem [49].

**Five Key Papers on HIV Viral Load in Resource-Limited Settings**

Majchrowicz, 2003 [10] One of the first articles written describing the scope of the problem.

Patton et al., 2006 [45] First description of the successful use of dried blood spots with the ultrasensitive p24 antigen assay.

Rouet et al., 2005 [62] First demonstration of real-time PCR technology for monitoring and diagnosis in a resource-limited country.

Schupbach et al., 1996 [15] An early paper comparing the ultrasensitive p24 antigen assay with HIV RNA viral load. Stevens et al., 2005 [25] First independent comparison of the ultrasensitive p24 antigen assay and the Cavidi reverse transcriptase assay in a resource-limited country.

Since this assay is newer than the Ultra p24 antigen assay, fewer data are available and most studies have been conducted either by or in collaboration with the manufacturer. Stevens and colleagues [25] compared the Roche RNA assay with both Ultra p24 antigen (external buffer) and the earlier version (version 1.0) of the Cavidi RT assay and found excellent correlation between RNA and RT results. The concentration of p24 antigen, RT, and RNA decreased in all patients after initiating ARV therapy, except in two individuals who had undetectable p24 and RT at baseline.

Crowe and colleagues have tested both the earlier version (version 1.0) and the more sensitive assay (version 2.0) in patients in Australia [49]. The version 1.0 RT assay had detectable HIV-1 RT present in 98% of samples (*n* = 127) with HIV-1 RNA >10,000 copies/ml. Sensitivity using the version 2.0 assay was higher, with detectable HIV RT in 95% of samples (*n* = 69) with HIV RNA >1,000 copies/ml. A positive association was found between the log_10_ HIV RNA copies/ml and log_10_ HIV RT fg/ml variables using Pearson correlation (*r* = 0.89, *p* < 0.001; *n* = 189 for version 1.0; *r* = 0.89; *n* = 85 for version 2.0). The RT activity closely followed the trend for HIV-1 RNA levels in samples (*n* = 4–10 per patient) from 10 HIV-1 infected patients with progressive disease [48]. Current (*n* = 40), previous (*n* = 30), or no (*n* = 119) exposure to efavirenz had only a minor effect on the RT assay despite the tight binding of efavirenz to the HIV-1 RT enzyme. There was a 0.20 log_10_ decrease in viral load in the samples with efavirenz resistance mutations, as measured by RT when compared to RT polymerase chain reaction (PCR), indicating a possible decrease in RT fitness [49].

Lombart and colleagues compared HIV-1 RNA (Roche COBAS Amplicor HIV-1 Monitor Test, version 1.5) with Ultra p24 antigen and the Cavidi RT assay, version 1.0 in samples from Burkina Faso [24]. The RT assay detected 0%, 93%, and 100% of samples with viral loads of <10,000, 10,000–63,000, and >80,000 copies/ml, respectively. Seyoum and colleagues [50] used the version 1.0 RT assay in a prospective study of 26 untreated patients with HIV in Ethiopia (subtype C), to compare results with the NucliSens QT assay. Although only 0.2 ml of plasma were used in this study instead of the 1.0 ml specified in the package insert, there was significant correlation between the two assays (*n* = 178, *r* = 0.65, *p* < 0.001), and patient RT activity in general mirrored HIV-1 RNA changes. Sivapalasingam and colleagues [51] compared results from the version 2.0 RT assay with the ultrasensitive Roche Amplicor Monitor HIV-1 RNA assay version 1.5 on plasma samples from patients in the United States (*n* = 29, subtype B) and Cameroon (*n* = 21, primarily CRF02_AG). They found that RT levels correlated significantly with plasma HIV-1 RNA viral loads using Spearman rank correlation (US: *r* = 0.89; *p* < 0.001; Cameroon: *r* = 0.67, *p* < 0.01). Among 32 samples with viral loads >2,000 copies/ml, 97% had detectable RT activity.

## Real-Time PCR/Molecular Beacon Assays

Real-time PCR such as TaqMan and Abbott RealTime, or molecular beacon assays such as Retina Rainbow or NucliSens EasyQ, might be useful for measuring viral load in resource-limited countries [52–65]. Real-time PCR detects amplicon production in real time with each PCR cycle, and thus does not rely on post-amplification detection of amplicons, which helps reduce the possibility of contamination and improves turnaround time. However, commercially available real-time PCR assays are just as expensive as the more standard nucleic acid viral load tests and also use expensive equipment. The use of in-house versions of these assays can help to reduce kit costs, but laboratories must provide their own reagents such as primers and probes, and optimize their methods. In settings with multiple HIV-1 subtypes, care must be taken in selecting reagents and amplification conditions, such as annealing temperatures. Quality assurance of each batch of such reagents remains problematic.

Although the NucliSens EasyQ assay has been evaluated in South Africa [58] and China [59], and the Abbott RealTime assay in Brazil [61], in general these assays do not provide a simple, less expensive alternative to viral load monitoring in resource-limited settings. Low-level contamination and a relatively high frequency of invalid results requiring repeat testing were some of the problems cited with these studies [58].

Many of these obstacles seem to have been overcome in Abidjan, Côte d’Ivoire, where TaqMan real-time PCR is being routinely used for infant diagnosis and patient monitoring [62]. The limit of quantitation for this assay was 300 copies/ml and RNA results were highly correlated with both the Versant (*n* = 327, *r* = 0.901, *p* < 0.001) and the Monitor (*n* = 101, *r* = 0.856, *p* < 0.001) HIV-1 RNA assay. The assay showed 100% sensitivity (*n* = 57) and specificity (*n* = 210) when used in the early diagnosis of infants compared with the Versant assay. The assay also proved useful for monitoring the response to treatment in 36 children and 46 adults who were initiating antiretroviral therapy.

## Solutions to Specimen Collection and Transport in Rural Areas

Viral load testing is typically performed on plasma, which requires a trained phlebotomist, access to a centrifuge, and a –70° C freezer for storage longer than a few days. Dried blood spots are an ideal alternative for specimen collection in rural areas because the blood is obtained either through a heel-stick or finger-stick and applied to filter paper. Once the blood has completely dried, it can be placed in its own gas impermeable ziplock bag along with dessicant and sent to the testing site at ambient temperature.

Dried blood spots have been used successfully to measure HIV-1 RNA in patients [66–73] and to diagnose perinatal HIV infection [45,73–87]. HIV-1 RNA in dried blood spots is stable for at least one year at room temperature [68,70] and HIV-1 DNA may be stable even longer [85,87], although proteins such as antibody and p24 antigen may be more labile [45,88]. Dried blood spots may also be used for HIV-1 subtyping [89] and genotypic resistance testing (S. Cassol, personal communication). A simplified extraction method used in conjunction with the Roche Amplicor DNA Test version 1.5 [85] or real-time PCR [57], and their use in the Ultra p24 antigen assay [45], makes dried blood spots an attractive way to diagnose infected infants early and get them into clinical care. Further options include the use of a new transport medium that stabilizes HIV-1 RNA in plasma [90] or the use of mobile laboratories in rural districts.

## What Is Needed for Implementation of Simplified Viral Load Testing?

First, each country must determine if the assay will quantify subtypes common in the region and is appropriate for the technical staff and laboratory equipment available. Infrastructure limitations impose significant barriers to implementation. Infrastructure includes both physical resources (water, reliable electricity, air conditioning, refrigeration, other equipment) and human resources (trained technologists). Training tools, trainers, guidelines, and consensus protocols, as well as monitoring and evaluation tools, including proficiency testing programs, all need to be established.

Costs must be taken into consideration—not only the costs of the kits, reagents, and supplies, but also the cost of equipment purchase (Does the assay require expensive equipment?), maintenance (Where is the nearest service technician located and how long does it take someone to repair a broken machine, and at what cost?), kit procurement (How long will it take to get kits from the manufacturer or distributor? Will lengthy delays in shipping or customs render the kits useless?), and the cost of labor (What are the hands-on time labor costs for the assay? Is local training available?). Other questions to consider include: Are there established external quality assurance programs available for the assay? Who will provide these? What will they cost? What approval is required by ministries of health?

Once the assay is deemed acceptable, clinical validation studies in relevant populations are required to prove comparability to the gold standard with respect to sensitivity, specificity, precision, reproducibility, dynamic range, and linearity. It is imperative that the laboratory performing the gold standard assay is participating in national or international quality assurance programs in order for the comparisons to be meaningful.

Lastly, there must be acceptance and understanding by the clinicians who will use the new assay. This will require involvement and education of clinicians with results from the clinical validation studies. Communication at a high level between those implementing laboratory monitoring tests and the clinicians advising government on the use of ARV therapy has been lacking. For example, there is no consensus regarding the level of monitoring that is required. Are ranges of values rather than exact values sufficient to provide inexpensive viral load monitoring? Clinician acceptance must play a role in devising a testing strategy for a given country. Endorsement by an international body such as the World Health Organization would likely increase the acceptability of these assays throughout the world.

## Conclusion

The currently available non-nucleic acid-based assays provide better options for resource-limited settings in terms of cost and physical requirements. However, they require basic laboratory infrastructure and as such do not meet the need for point-of-care tests for patients without access to central or district hospitals. Much simpler tests, based on dip-stick technology [91] or molecular zipper assays [92], are in development. It is hoped that data describing their applicability will be available soon.

## Abbreviations

ARV: antiretroviral
ELISA: enzyme-linked immunosorbent assay
PCR: polymerase chain reaction
RT: reverse transcriptase
